# Extraocular spread of uveal melanoma to the breast: a rare clinical presentation

**DOI:** 10.1093/jscr/rjag587

**Published:** 2026-09-14

**Authors:** Isabella H Muti, Meghan Maceyko, Reem Khraishi, Juan Palazzo, Alliric I Willis

**Affiliations:** Sidney Kimmel Medical College, Thomas Jefferson University, 1025 Walnut Street, Philadelphia, PA 19107, United States; Department of Surgery, Division of Surgical Oncology, Thomas Jefferson University Hospital, 1100 Walnut Street, Philadelphia, PA 19107, United States; Department of Pathology and Genomic Medicine, Thomas Jefferson University Hospital, 1020 Locust Street, Philadelphia, PA 19107, United States; Department of Pathology and Genomic Medicine, Thomas Jefferson University Hospital, 1020 Locust Street, Philadelphia, PA 19107, United States; Department of Surgery, Division of Surgical Oncology, Thomas Jefferson University Hospital, 1100 Walnut Street, Philadelphia, PA 19107, United States

**Keywords:** uveal melanoma, metastatic melanoma, breast cancer

## Abstract

Uveal melanoma (UM) is a rare malignancy with a high risk of delayed metastasis, primarily to the liver. Metastatic spread to the breast is exceedingly uncommon and may mimic primary breast carcinoma, posing diagnostic challenges. We report the case of a patient with a history of UM who was found to have a new right breast lesion on surveillance magnetic resonance imaging 18 years after initial UM diagnosis. The patient underwent Strut-Adjusted Volume Implant Scout guided partial mastectomy. Histopathology of the surgical specimen confirmed metastatic UM. This case underscores the unpredictable metastatic behavior of UM and highlights the importance of maintaining clinical vigilance and a broad differential diagnosis, even decades after initial treatment.

## Introduction

Uveal melanoma (UM) is an uncommon malignancy, accounting for approximately 3%–5% of all melanoma cases in the United States [1]. It predominantly affects older adults, with the median age at diagnosis of 62 years, and is most frequently seen in individuals of Caucasian descent with fair skin and light-colored eyes [2]. Although primary treatment is often effective at achieving local control, UM is characterized by a high propensity for delayed metastases via hematogenous dissemination. The liver is the most common site of metastasis, involved in up to 90% of cases, followed by the lungs, bone, skin, and subcutaneous tissue [3]. Once metastatic, prognosis is poor with an estimated median survival of 10–13 months [3, 4].

Metastatic spread of UM to the breast is exceptionally rare, with only a limited number of cases reported in the literature [5]. When present, it typically reflects disseminated disease rather than an isolated or early site of metastasis [3]. Further, since breast metastasis may clinically and radiographically mimic primary breast carcinoma, it often poses a diagnostic challenge.

We report a case of UM metastasis to the breast 18 years after the patient’s initial diagnosis. Both the unusually long disease-free interval and the breast as the presenting site of recurrence underscore the unpredictable metastatic behavior of UM and highlight the importance of maintaining clinical vigilance and a broad differential diagnosis, even decades after initial treatment.

## Case report

A 59-year-old female presented with a new diagnosis of UM metastatic to the breast, identified on surveillance imaging. She had a prior history of Stage IIa (cT2a, cN0, cM0) choroidal melanoma of the right eye, diagnosed eighteen years earlier. At the time of diagnosis eighteen years earlier, the uveal lesion measured 13 × 8 mm in basal diameter with a thickness of 5.3 mm. Genetic testing at the time revealed monosomy 3. She was treated at that time with radioactive iodine plaque therapy, followed by 1 year of adjuvant sunitinib.

**Figure 1:**
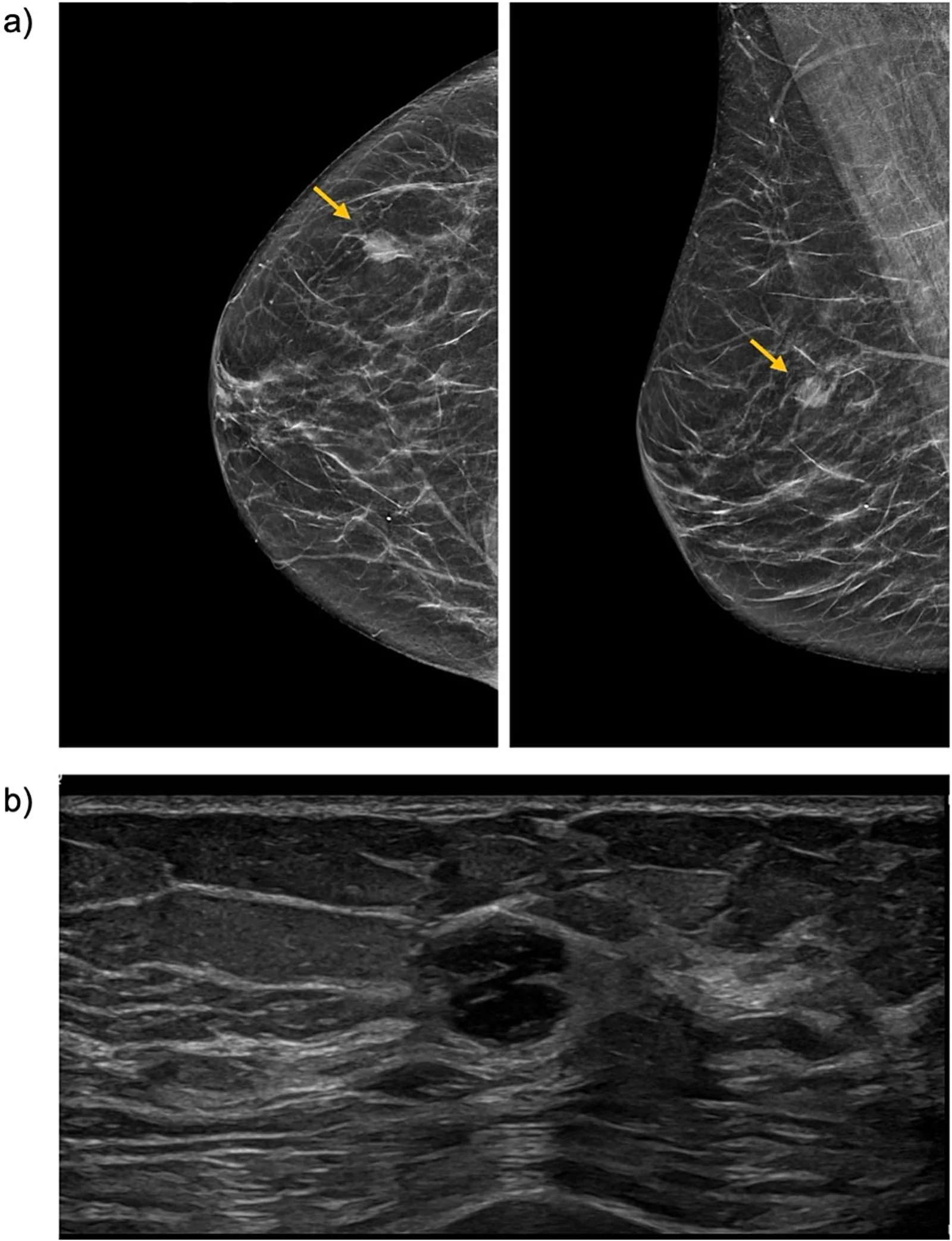
(a) Mammographic images showing 1 cm oval mass in the outer right breast. (b) Ultrasound of hypoechoic oval cystic and solid mass with posterior acoustic enhancement measuring 1.0 × 0.7 × 0.8 cm.

The patient subsequently underwent long-term surveillance consisting of annual chest radiography and abdominal magnetic resonance imaging (MRI). She initiated routine screening mammography at age 40. Eighteen years after her initial diagnosis, surveillance abdominal MRI revealed a 9 mm, T2 hyperintense lesion within the right breast. A screening mammogram performed ten months earlier had no suspicious findings. Subsequent diagnostic mammography and targeted breast ultrasonography demonstrated a new hypoechoic, oval cystic mass with posterior acoustic enhancement and microlobulated borders measuring 1.0 × 0.7 × 0.8 cm in the outer right breast, 5 cm from the nipple, confirmed not seen on prior imaging (Fig. 1). Ultrasound-guided core needle biopsy confirmed metastatic melanoma. Subsequent staging with positron emission tomography and computed tomography (PET-CT) and brain MRI showed no evidence of metastatic disease outside the breast.

**Figure 2:**
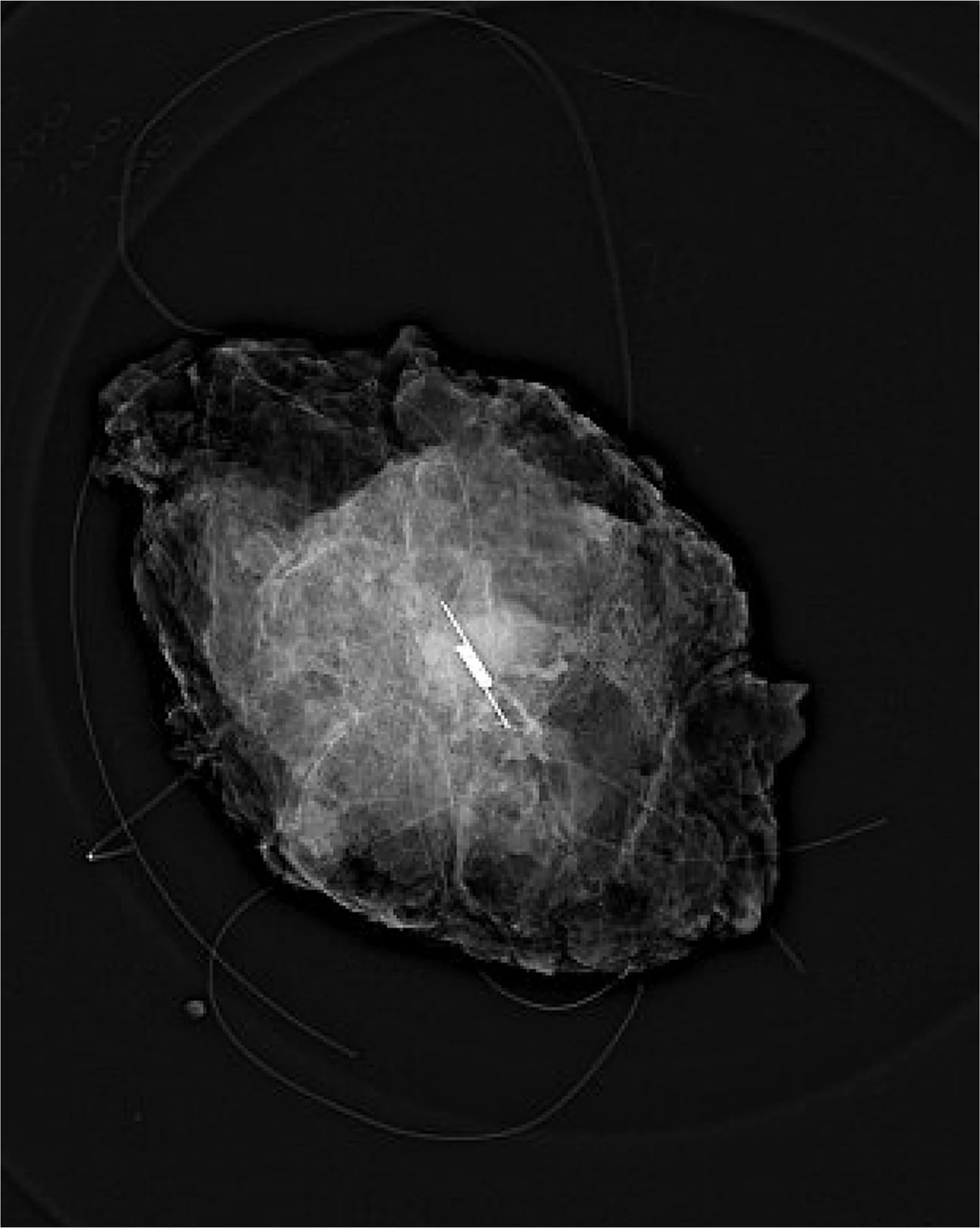
Mammographic image showing surgical specimen from the right breast excised using SAVI scout guidance.

**Figure 3:**
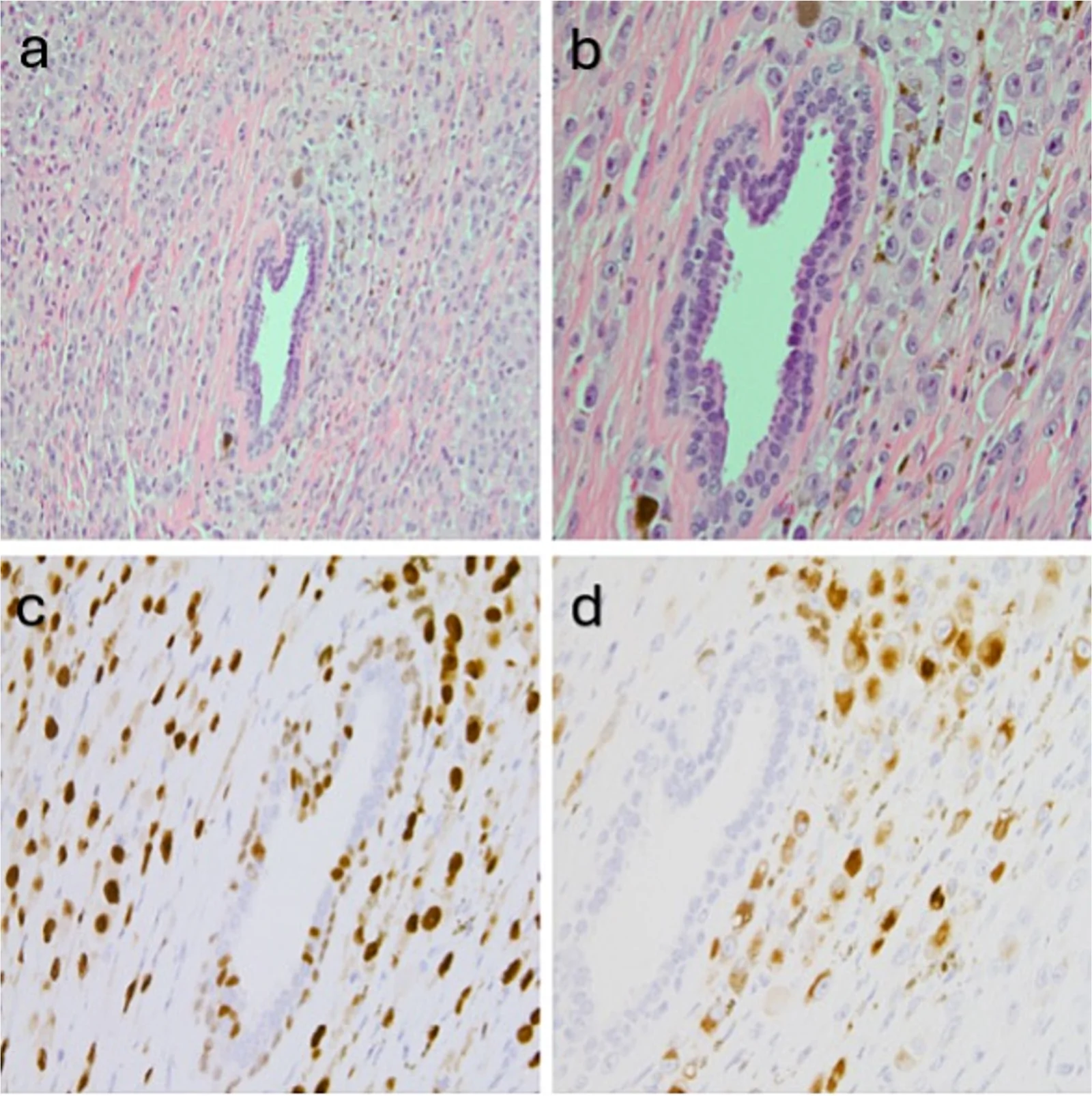
(a) H&E, 20×: Infiltrative solid growth of melanocytic cells surrounding a benign breast duct with scattered melanin pigment. (b) H&E, 40×: Higher power shows tumor cells with large pleomorphic nuclei and prominent nucleoli. Tumor cells show diffuse nuclear positivity for SOX10 (c) and positive cytoplasmic staining for Melan-A (d), supporting the diagnosis of metastatic melanoma involving the breast.

The patient underwent a Strut-Adjusted Volume Implant (SAVI) Scout-guided partial mastectomy of the right breast with negative margins (Fig. 2). Histologic examination demonstrated an infiltrative solid growth of melanocytic cells with large pleomorphic nuclei, prominent nucleoli, and scattered melanin pigment. Immunohistochemical staining was positive for SOX10 and Melan-A, and negative for AE1/AE3 and S100, supporting the diagnosis of metastatic melanoma (Fig. 3).

## Discussion

UM is biologically distinct from cutaneous melanoma and is characterized by an unpredictable metastatic course with the potential for long periods of clinical dormancy [6]. Although local control is commonly achieved with eye-directed therapy, up to half of patients will ultimately develop metastatic disease, often years after initial diagnosis [7]. The present case is notable for an exceptionally prolonged disease-free interval of 18 years and for the breast as the sole site of metastatic recurrence, emphasizing the enduring metastatic risk associated with UM.

Both tumor thickness and presence of monosomy 3 are well-established prognostic markers associated with increased risk of metastatic spread in UM [8]. Current National Comprehensive Cancer Network guidelines recommend more intensive imaging surveillance for up to ten years following initial treatment in patients with high-risk features, including monosomy 3 [9]. The patient described here continued surveillance imaging beyond the recommended period, underscoring that even patients with prolonged intervals of apparent remission remain at risk for delayed recurrence. However, the optimal duration and scope of surveillance remain undefined and uncommon sites of metastasis are not routinely evaluated, highlighting the limitations of standard imaging surveillance.

Despite its overall rarity, melanoma has been reported to be the most common extramammary malignancy to metastasize to the breast. When present, metastatic lesions may mimic benign breast findings or primary breast carcinoma on imaging. Breast involvement also frequently occurs in the setting of disseminated disease [10–12]. Spread of UM to the breast, *however*, is exceedingly uncommon and represents only a small fraction of documented non-mammary breast metastases [5, 10, 12]. In most cases, metastatic tumors are histologically consistent with the primary tumors [10, 11]. Histopathologic evaluation is therefore essential to differentiate metastatic disease from primary breast cancer.

In this case, the metastatic lesion was incidentally detected within the breast on abdominal MRI with benign-appearing features, emphasizing the importance of clinical context and tissue diagnosis. Fortunately, the patient had been consistent with breast cancer screening mammograms such that recent breast imaging demonstrated no mass less than 1 year prior. Therefore, diagnostic evaluation and biopsy were immediately pursued. Histopathologic evaluation with immunohistochemistry remains essential, as metastatic melanoma may resemble poorly differentiated breast carcinoma. Positivity for SOX10 and Melan-A with absence of epithelial markers supported the diagnosis of metastatic melanoma in this patient [12].

There is no consensus standard of care for isolated extrahepatic metastases from UM. Surgical resection may be considered in carefully selected patients with oligometastatic disease, as in this case, where negative margins were achieved. Systemic treatment decisions are individualized, and emerging therapies may offer benefit in selected patients, such as those with HLA-A*02:01 phenotype [13], though overall prognosis remains unclear.

In conclusion, this case highlights the prognostic significance of primary tumor features such as tumor thickness and monosomy 3, demonstrates the capacity for UM to recur after more than a decade of dormancy, and underscores the need for sustained clinical awareness and individualized surveillance strategies long after initial treatment.
